# Sequence variation, evolutionary constraint, and selection at the *CD163* gene in pigs

**DOI:** 10.1186/s12711-018-0440-8

**Published:** 2018-12-20

**Authors:** Martin Johnsson, Roger Ros-Freixedes, Gregor Gorjanc, Matt A. Campbell, Sudhir Naswa, Kimberly Kelly, Jonathan Lightner, Steve Rounsley, John M. Hickey

**Affiliations:** 1 The Roslin Institute and Royal (Dick) School of Veterinary Studies, The University of Edinburgh, Midlothian, EH25 9RG Scotland, UK; 2 Department of Animal Breeding and Genetics, Swedish University of Agricultural Sciences, Box 7023, 750 07 Uppsala, Sweden; 3 Genus plc, 1525 River Road, DeForest, WI 53532 USA

## Abstract

**Background:**

In this work, we investigated sequence variation, evolutionary constraint, and selection at the *CD163* gene in pigs. A functional CD163 protein is required for infection by porcine reproductive and respiratory syndrome virus, which is a serious pathogen with major impacts on pig production.

**Results:**

We used targeted pooled sequencing of the exons of *CD163* to detect sequence variants in 35,000 pigs of diverse genetic backgrounds and to search for potential stop-gain and frameshift indel variants. Then, we used whole-genome sequence data from three pig lines to calculate: a variant intolerance score that measures the tolerance of genes to protein coding variation; an estimate of selection on protein-coding variation over evolutionary time; and haplotype diversity statistics to detect recent selective sweeps during breeding.

**Conclusions:**

Using a deep survey of sequence variation in the *CD163* gene in domestic pigs, we found no potential knockout variants. The *CD163* gene was moderately intolerant to variation and showed evidence of positive selection in the pig lineage, but no evidence of recent selective sweeps during breeding.

## Background

In this work, we investigated sequence variation, evolutionary constraint, and selection at the *CD163* gene in pigs. A functional CD163 protein is required for infection by porcine reproductive and respiratory syndrome virus (PRRSV) [1], which is a serious pathogen with major impacts on pig production [2]. PRRSV-resistant genome-edited pigs with a modified *CD163* gene have been developed, either by knocking out the gene completely or by targeting its fifth scavenger receptor cysteine-rich (SRCR) domain, which is essential for virus PRRSV infection [3–6].

The physiological functions of the CD163 protein include clearing haemoglobin from blood plasma [7], adhesion of nucleated red blood cells to macrophages during red blood cell differentiation [8], and immune signalling [9–11]. When red blood cells rupture, and haemoglobin is released into the blood stream, it is bound by haptoglobin, and the haptoglobin—haemoglobin complex is taken up by macrophages using the CD163 protein as receptors on their surface [7]. Since the natural function of CD163 is receptor-mediated endocytosis, it is a target for pathogens entering cells. At least one other virus, the simian hemorragic fever virus [12], has independently evolved to target CD163.

Given that genome editing of *CD163* has led to PRRVS-resistant pigs, we wanted to determine if natural knockout variants for the *CD163* gene could be identified in elite pigs, in order to investigate the opportunity to select for resistance to PPRSV within existing breeding programs. The aims of this paper were to survey *CD163* sequence variation for such naturally occurring knockout variants (i.e., stop-gain and frameshift variants that likely disrupt gene function), and to compare the *CD163* sequence variation to genomic distributions of selection and constraint. We used targeted pooled sequencing of *CD163* exons to detect sequence variants in 35,000 pigs of diverse genetic backgrounds. Then, we used whole-genome sequence data from three pig lines to put these results of *CD163* sequence variation in the context of the whole genome. We used three complementary population genetic analyses: a variant intolerance score, which measures the tolerance of genes to protein coding variation; a selection test on protein coding variation over evolutionary time; and haplotype diversity statistics to detect recent selective sweeps during breeding.

## Methods

### Data

We used targeted *CD163* exon sequence data and whole-genome sequence data from pigs in the Pig Improvement Company (PIC) breeding programme. This programme contains a diverse collection of genetics, which represent broadly used populations, including animals of Large White, Landrace, Duroc, Hampshire and Pietrain heritage. The targeted sequencing included DNA samples from 35,000 pigs, which were previously collected from 2011 to 2016 as part of the breeding programme.

To put the targeted exon sequence data in a genomic context and compare it to genomic distributions of selection and constraint, we used whole-genome sequence data from three lines of pigs of the PIC breeding programme. These lines were also sampled in the targeted exon sequencing. We used 1146 individuals from Line 1, sequenced at various coverages. Eighty-four individuals were sequenced at 30X coverage, 11 at 10X, 45 at 5X, 561 at 2X, and the remaining 445 at 1X. Individuals and their sequence coverages were chosen with the AlphaSeqOpt algorithm [13, 14], and we added sires that contributed a large proportion of the progeny in Line 1 that were genotyped as part of the routine breeding activities of PIC. AlphaSeqOpt uses phased genotype data, which in our case consisted of single nucleotide polymorphism (SNP) genotypes from a 60 K or 15 K SNP chip, which were phased with AlphaPhase [15]. The AlphaSeqOpt algorithm consists of several steps. First, we identified focal individuals that had a genome representing the haplotype diversity of the population as much as possible, and allocated a fixed sequencing budget to the families of the focal individuals in order to maximise phasing accuracy proportionate to the population haplotype footprint of the focal individual. Then, we identified individuals that carried underrepresented haplotypes to maximise the number of haplotypes that were sequenced at sufficiently high coverage for accurate imputation. We also used 408 individuals from Line 2 and 638 individuals from Line 3, which were all sequenced at 2X coverage. These individuals were sires that contributed a large proportion of the progeny in Lines 2 and 3 that were genotyped as part of the routine breeding activities of PIC.

### Targeted sequencing of *CD163*

We used a hierarchical pooling strategy to sequence *CD163* exons in many individuals cost-effectively. Using sequence capture, we could target the sequencing effort to the *CD163* exons only, and thus fit many animals into the same lane of an Illumina sequencer. The pooling allowed us to use fewer targeted capture reactions, while retaining the ability to go back to the original plate and individually sequence animals for validation.

Therefore, we pooled 96 DNA samples each into one combined DNA sample and constructed a shotgun sequencing library using the ThruPLEX Tag-seq kit from Rubicon Genomics. This kit incorporates unique molecular identifiers that allow a consensus sequence to be generated from reads originating from the same molecule, and thus reduces the impact of sequencing errors. Twenty-four such barcoded libraries were combined and used as input into a sequence capture reaction with baits that were designed against the *CD163* exons (Arbor Biosciences, Ann Arbor, MI). Then, the product of the library capture was used to generate 2 × 150-bp reads on an Illumina MiSeq sequencer. This pooling scheme allowed us to sequence up to 2304 samples per sequencing run. In total, 35,808 animals were sequenced using this scheme.

We aligned reads with BWA (v 0.7.15-r1140) [16], using the BWA-MEM algorithm, against the 10.2 version of the pig genome to which we added a 33-kb contig representing the *CD163* genomic region that was missing from this version of the reference genome. The coding sequence of *CD163* on this contig is identical to the sequence that is annotated as *CD163* in the version 11.1 of the pig reference genome. We used Connor (https://github.com/umich-brcf-bioinf/Connor) to call consensus sequences from reads with the same unique molecular identifier and called variants from these consensus alignments using the LoFreq variant caller [17]. We used snpEff [18] to classify the variants as synonymous, nonsynonymous, stop-gain and frameshift indel variants.

### Validation of potential knock-out variants

Potential stop-gain variants detected in the pooled targeted sequencing data were pursued for validation by sequencing individual animals, i.e. we went back to the pools in which the variants were detected and sequenced amplicons of the appropriate exons from all the DNA samples that made up the pool with individual barcodes on the MiSeq. None of the potential stop-gain variants were validated in the individual sequencing. We tested five potential frameshift indel variants in the same way, and none of these were validated in the individual sequencing either.

### Whole-genome sequence data processing

We aligned reads to the pig genome (Sscrofa11.1) with BWA-MEM [16], removed duplicates with Picard (https://broadinstitute.github.io/picard/index.html), and called variants with the GATK HaplotypeCaller [19]. We filtered and processed variant call format files with VCFtools [20]. We used the Ensembl variant effect predictor [21] to find the protein-coding SNPs, and classify them into synonymous and nonsynonymous SNPs based on the Ensembl gene annotation [22] version 90. We downloaded variants in *CD163* from the Ensembl variation database.

### Residual variant intolerance score

The residual variant intolerance score [23] measures gene-level tolerance to mutations by counting segregating variants. To calculate the residual variant intolerance, we counted the number of nonsynonymous variants and the total number of variants in each gene, and calculated the studentised residual of the regression between the number of nonsynonymous variants and the total number of variants. We included variants that segregated in at least one of the three lines. We applied the residual variant intolerance score both at the level of the gene, and at the level of the protein domain [24], using protein domains that were found by identifying Pfam profiles in Ensembl protein sequences with PfamScan [25]. All gene-level analyses were performed on the principal transcript, as designated with APPRIS annotation [26].

### Selection analysis in the pig lineage

SnIPRE [27] uses a Poisson model to measure gene-level selection based on between-species divergence and within-species polymorphism. We calculated the divergence between the pig and cattle (UMD 3.1.1) genomes using the Nei-Gojobori method [28], which estimates the number of potential synonymous and nonsynonymous substitutions between two codons. We aligned the reference genomes using Lastz [29] and refined the alignments using the chain/net method [30]. We excluded all codons that were not fully aligned between genomes, i.e., any codon that contained an alignment gap or a missing base in any of the genomes. For within-species polymorphism data, we used the protein-coding variants from whole genome sequence data of the three lines combined.

SnIPRE models the logarithm of the mutation count in a sample of individuals as a linear function of fixed effects (an intercept term, a term for nonsynonymous variants, a term for divergent variants, an interaction term for divergent nonsynonymous variants, and an offset term) and random gene effects, which allow the coefficients for nonsynonymous variants, divergent variants and the interaction between them to be estimated with regularisation. The selection effect for each gene is the interaction term for nonsynonymous fixed variants (summing the coefficient for the fixed effect and the gene-specific coefficient for that gene), i.e. it provides an estimate of the excess or deficit in nonsynonymous divergent variants in a gene. We ran the empirical Bayes implementation of SnIPRE, using the lme4 R package [31], which generates confidence intervals for the selection effect based on standard errors.

### Selective sweep analysis by haplotype diversity

We estimated haplotype diversity at *CD163*, at 100 random control genes of similar length as *CD163* (at most 10% difference), and at 11 homologs of genes that are stably expressed in humans [32]. We imputed genome-wide sequence data to 65,000 pigs from Line 1, using SNP genotypes from a 60 K or 15 K SNP chip and the Line 1 sequence data described above. We extracted mapped read counts that supported each allele from low coverage samples, as outlined in [33], and used multilocus hybrid peeling [34], as implemented in AlphaPeel, to phase and impute all individuals to full sequence data in the selected genes.

We extracted all variants that were within exons and introns of the genes and identified the haplotypes that were carried by each genotyped individual in each gene. For each gene, including introns, strings of phased alleles were compared to define haplotypes carried by each individual in each parental chromosome. Strings of alleles that were identical (with a mismatch threshold) between two individuals were considered to be the same haplotype, while strings with more than two mismatches were considered as different haplotypes to account for sequencing or phasing errors. Then, we calculated haplotype homozygosities based on the pooled frequency of the two most common haplotypes (H_12_), which is used as a test statistic for detection of selective sweeps, and has been shown to be sensitive to soft sweeps [35].

### Gene ontology enrichment of gene lists

We downloaded gene ontology (GO) biological process terms for all Ensembl genes from BioMart [36], and ranked enriched biological processes based on p values from a Fisher’s exact test of independence. For comparison, we extracted the genes found to be under positive selection in the pig, based on dN/dS ratio in [37], and mapped their names to Ensembl gene identifiers with BioMart. We found enriched biological process terms in this gene list in the same way as in our data, and identified the overlap of genes with enriched gene ontology terms between the two lists.

## Results

### Identification of *CD163* sequence variants

**Fig. 1:**
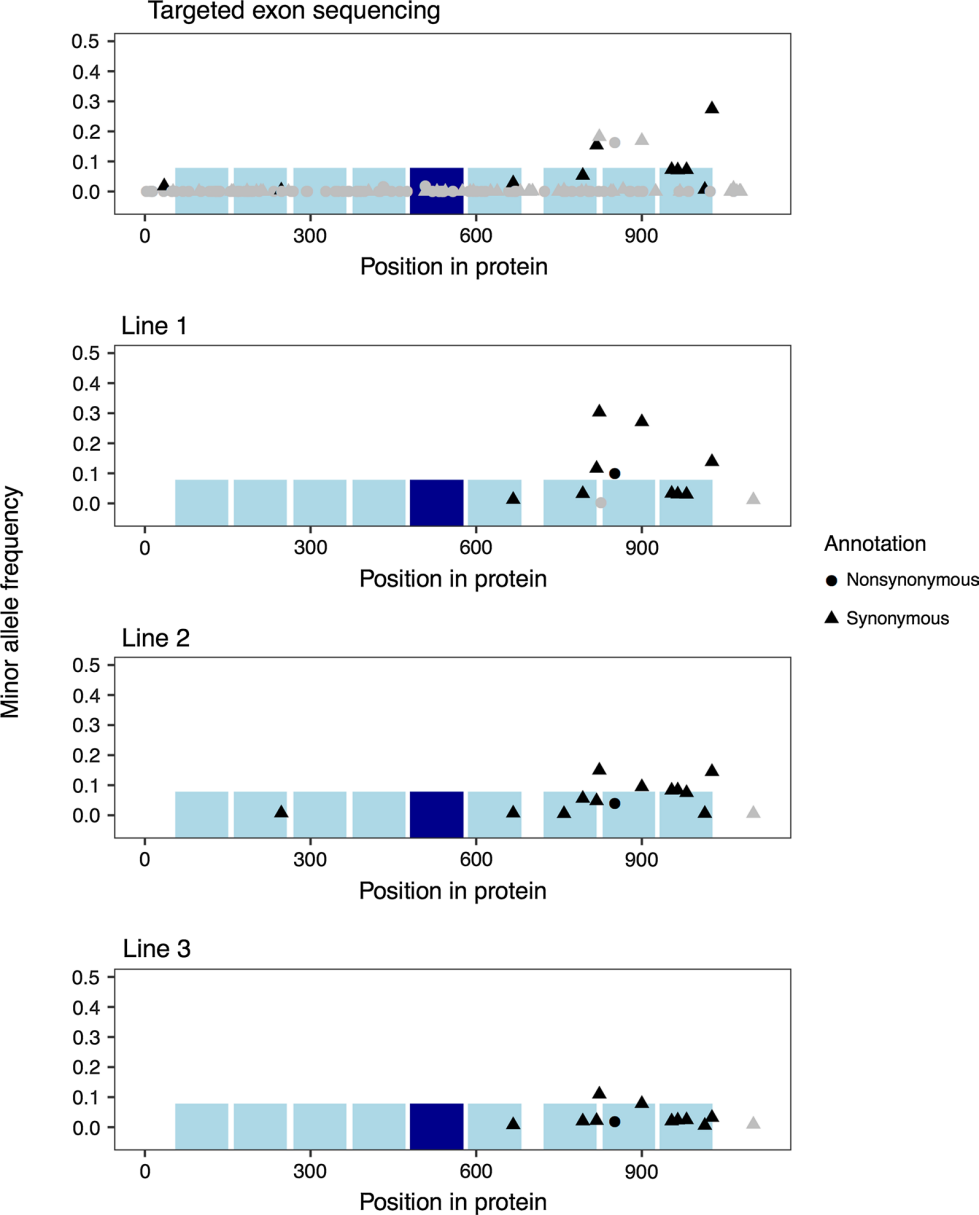
Protein-coding SNPs in *CD163* identified by targeted exon sequencing and whole-genome sequencing of three pig lines. The horizontal axis indicates the position of the variant in the amino acid sequence of the protein. The vertical axis is the minor allele frequency, which shows that discordant variants are rare. Grey and black coloured points indicate replication. Grey dots in targeted sequencing are variants that are not present in the Ensembl variation database. Grey dots in the whole-genome sequenced lines are variants that are not replicated by the targeted exon sequencing. The blue boxes represent SRCR domains, with the fifth domain (which is the target of the PRRS virus) in dark blue

We used a hierarchical pooling strategy to sequence the exons of *CD163* from over 35,000 pigs from nine lines and identified *CD163* variants from whole-genome sequencing of 1146, 638, and 408 pigs from three of these nine lines. Targeted sequencing of exons identified 140 single nucleotide variants in *CD163*. Whole-genome sequencing in three of the nine lines identified 15 single nucleotide variants in *CD163*, two of which were nonsynonymous, the rest being synonymous, and no potential knockout variants. Table 1 shows the numbers of synonymous and nonsynonymous single nucleotide variants found in each dataset, and the overlap between them. Figure 1 shows the location of the variants in the CD163 protein sequence and their frequencies in targeted and whole-genome sequencing. The targeted sequencing also identified 14 potential stop-gain variants, which all occurred at low frequency (mean 0.01%; maximum 0.4%). We further investigated these stop-gain variants by performing individual sequencing of the animals that composed the pool in which the potential stop-gain variant was identified. This ruled out all potential stop-gain variants as false positives that were likely caused by polymerase errors during amplification before incorporation of unique molecular identifiers. The targeted sequencing also identified 45 potential frameshift indel variants, which all occurred at low frequency (mean 0.03%; maximum 0.1%). We further investigated five of these variants by individual sequencing and ruled them out also as false positives.

**Table 1:** Number of pairwise shared SNPs between sets of variants identified in *CD163* based on targeted sequence of nine lines and based on whole-genome sequence of three of these lines (Lines 1, 2, and 3)

	Synonymous				Nonsynonymous			
	Targeted	Line 1	Line 2	Line 3	Targeted	Line 1	Line 2	Line 3
Targeted	49	9	12	10	91	1	1	1
Line 1		10	10	11		2	1	1
Line 2			13	10			1	1
Line 3				11				1

The *CD163* variants detected in the whole-genome sequence data of three pig lines were mostly concordant with those from targeted exon sequencing. The discordant SNPs found in the whole-genome sequence data had low allele frequencies. One nonsynonymous shared variant, K851R, occurred at a relatively high minor allele frequency (16% in the targeted exon sequencing, 10% in Line 1, 4.0% in Line 2, and 1.9% in Line 3) and is located in the eighth SRCR domain. Among the sequence variants detected, 10 were already present in the Ensembl variation database. These were all synonymous and had a higher minor allele frequency (mean equal to 7.6%) than variants that were missing from Ensembl variation (mean minor allele frequency 0.44%).

### Residual variant intolerance score

**Fig. 2:**
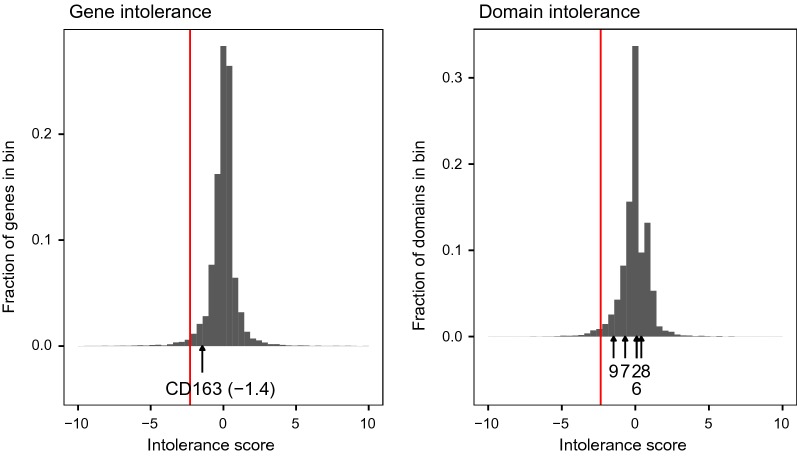
Distributions of residual variant intolerance scores across the pig genome. The red line is the 2% percentile of the distribution. Arrows indicate the position of *CD163* and five of its SRCR domains

*CD163* was not among the most variant-intolerant genes based on the residual variant intolerance score. Figure 2 shows the distributions of gene-level and protein domain-level residual variant intolerance scores with the ranking of *CD163* and its five variable domains. *CD163* ranked as number 894 out of 17,982 variable autosomal genes based on the residual intolerance score, while the five variable SRCR domains of *CD163* ranked as numbers 1037 (domain 9), 2686 (domain 7), 8125 (domains 2 and 6), and 14,147 (domain 8) out of 19,930 variable protein domains, as measured by the residual variant intolerance score applied to protein domains identified with the Pfam database.

**Fig. 3:**
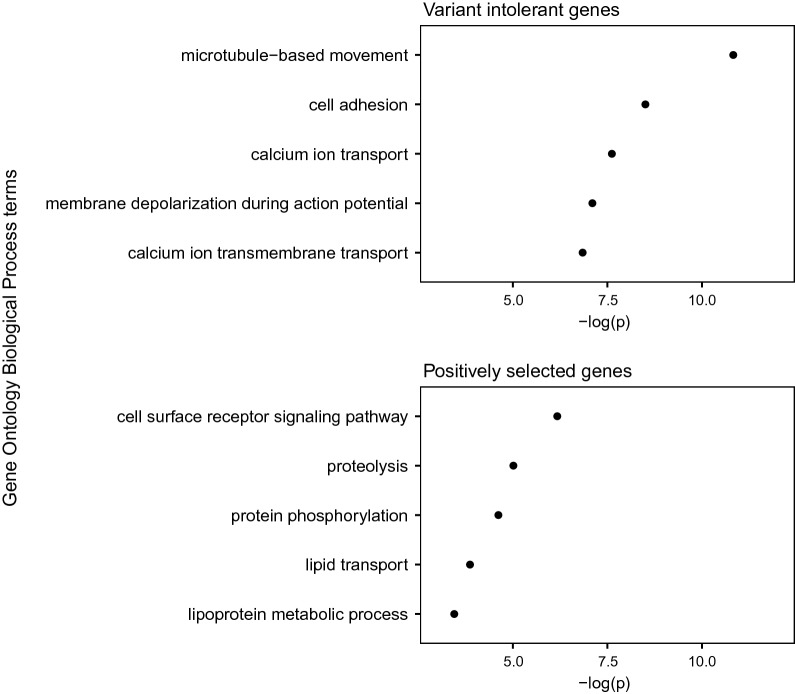
The five most enriched gene ontology biological process terms in variant-intolerant genes and positively selected genes in the pig, with the negative logarithm of the p value of their enrichment based on Fisher’s exact test

We used the bottom 2% of the genome-wide residual variant intolerance distribution to identify 358 variant-intolerant genes. The list was enriched for basal cellular processes such as microtubule-based movement, cell adhesion, and calcium ion transport (Fig. 3). Conversely, the top 2% of the residual variant intolerance score distribution was enriched for olfaction-related and immunity-related terms, namely G-protein coupled receptor signalling, detection of chemical stimulus involved in sensory perception of smell, antigen processing and presentation, and response to stimulus.

### Positive selection in the pig lineage

**Fig. 4:**
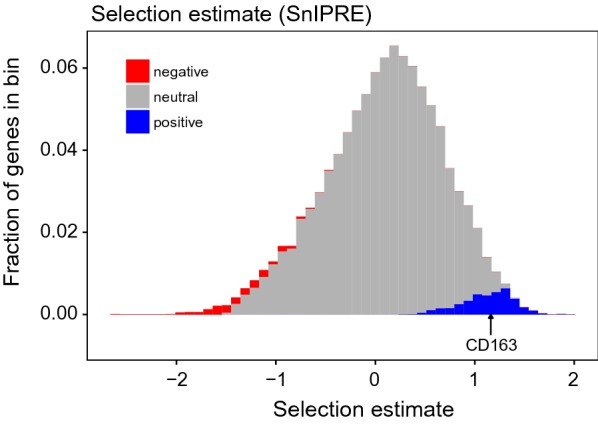
SnIPRE estimates of selection. The arrow indicates the position of *CD163*, which is one of the potentially positively selected (blue) genes

*CD163* showed evidence of positive selection in the pig lineage, as estimated by the SnIPRE model. Figure 4 shows the selection estimates from the SnIPRE model, highlighting positively and negatively selected genes and the position of *CD163*. We found 1125 putatively selected genes, 778 positively selected genes, and 347 putatively negatively selected. Positively selected genes in the pig lineage were enriched for cell surface receptor signalling, proteolysis, protein phosphorylation and terms related to lipids (Fig. 3).

There was limited overlap between the positively selected genes in our study and those reported by Groenen et al. [37]. We were able to map 287 of the 331 positively selected genes from the previous study to Ensembl gene identifiers, and nine of these genes were shared with our list of positively selected genes. However, both of the gene lists were enriched for the GO term ‘protein phosphorylation’ (at Fisher’s test p values < 0.001).

### Selective sweep analysis

**Fig. 5:**
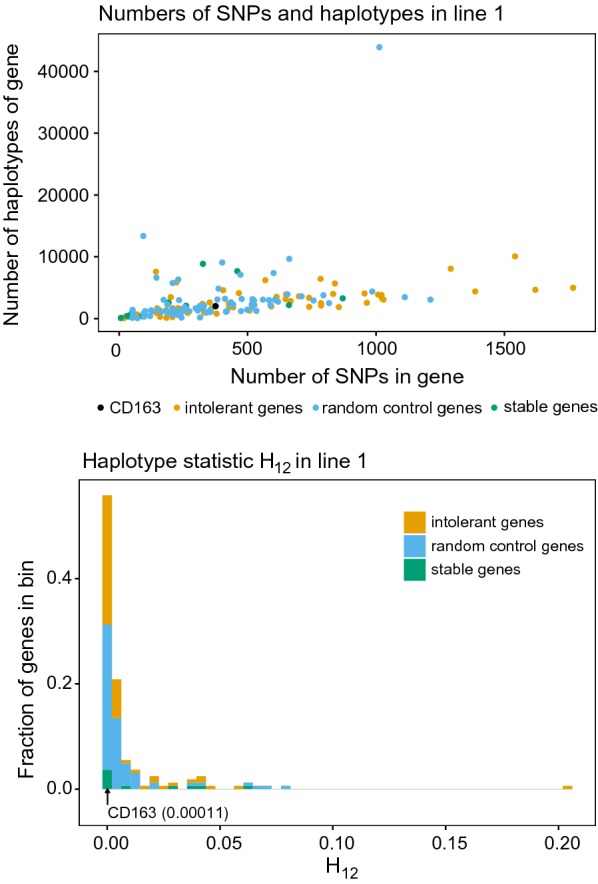
Number of haplotypes, number of SNPs per gene, and selective sweep statistic H_12_ for *CD163*, 100 control genes of similar length, 100 intolerant genes with a low residual variant intolerance score, and 11 control genes that are homologs of human genes with stable expression across many tissues

We investigated haplotype diversity of *CD163* in one of the lines using imputed whole-genome sequence data. We calculated the selective sweep test statistic H_12_ for *CD163*, and compared it to that for 100 randomly selected control genes of similar length. Figure 5 shows H_12_ at *CD163*, the 100 control genes, a set of homologs of genes that are stably expressed in humans, and randomly selected genes labelled as intolerant based on the residual variant intolerance score. Based on this, *CD163* showed no evidence of a recent selective sweep.

## Discussion

In this paper, we investigated sequence variation, evolutionary constraint, and selection at the *CD163* gene in pigs. Within *CD163*, we identified synonymous and nonsynonymous variants but no potential knockout variants. We found that *CD163* is relatively tolerant to variation, shows evidence of positive selection in the pig lineage, but no evidence of selective sweeps during recent breeding. In light of these results, we will discuss (1) variant intolerance scores; (2) selection on *CD163* in the pig lineage (3) the lack of evidence of recent selective sweeps; and (4) some technical aspects of the targeted exon sequencing method.

### Residual variant intolerance score

Variant intolerance scores measure the lack or excess of common nonsynonymous variants in a gene [23]. A low variant intolerance score for a gene indicates that its sequence is constrained, which correlates with gene essentiality [38]. The intermediate variant intolerance scores of *CD163* suggest that it is moderately constrained in the pig. Given the known functions of *CD163* in haemoglobin scavenging and immune signalling [7–11], it appears that *CD163* is under purifying selection, but not as strongly constrained as essential genes involved in basic cellular functions.

The 2% bottom extreme tail of the variant intolerance distribution was enriched for genes that are related to microtubule-based movement, which is consistent with an enrichment of microtubule-genes in human essential genes [38]. The 2% top extreme tail of the variant intolerance distribution was enriched for genes that are related to olfaction and antigen processing and presentation, which is again consistent with results from humans [23], where olfactory receptors and human leukocyte antigen genes tend to have high residual variant intolerance scores.

### Selection in the pig lineage

The SnIPRE model is a generalized mixed linear model that estimates the selection effect on each gene based on the number of fixed nonsynonymous substitutions compared to an outgroup species [27], for which we used cattle. A positive selection estimate is based on a significantly larger number of nonsynonymous fixed substitutions when comparing the porcine and the bovine genomes than expected under neutrality, which is assumed to hold for synonymous sites. The positive selection effect estimated for *CD163* suggests that its sequence is quite flexible and has been subjected to accelerated evolution in the pig lineage. Positive selection on *CD163* is consistent with its known role in infection. The estimated positive selection effect for other cell surface genes, including those encoding the T cell surface proteins *CD3*, *CD5* and *CD8A* and immunoglobulin receptors *FCER1A* and *FCGR1A*, is consistent with previous observations of selection on immune genes in the pig [37]. However, the overlap between genes found to be positively selected here and in Groenen et al. [37] is limited.

### Selective sweep analysis

Selective sweeps occur when fixation of one or more beneficial variants affects allele frequencies at linked sites [39]. Such signals of recent selection (within ≪ *N*_*e*_ generations) can be detected from population genetics data. When the beneficial variant is already present in the population as standing variation, selection may give rise to a so-called soft sweep, which may be more difficult to detect than a sweep that arises from a beneficial new mutation [40]. Since selection on standing variation is the expectation in animal breeding, we used a statistic designed to detect soft sweeps [35]. The lack of a selective sweep at *CD163* suggests that it has not been a target of strong recent selection during pig breeding, which is consistent with its lack of obvious connections to traits that are under strong artificial selection, such as production and reproduction traits. However, selective sweep analysis cannot rule out the possibility that *CD163* variants could have small effects on some quantitative trait and may have been subjected to subtle allele frequency shifts by selection.

### Technical aspects of the targeted exon sequencing

Targeted exon sequencing of pooled samples is a feasible way to sequence a gene in many individuals cost-efficiently. However, as our unsuccessful validation of potential stop-gain and frameshift variants shows, this method suffers from low-frequency false positives, which are likely due to polymerase errors before incorporation of unique molecular identifiers. This suggests that the low-frequency nonsynonymous variants detected in the targeted exon sequencing are also likely to be false positives. The targeted exon sequencing and whole-genome sequencing were in good agreement for higher frequency variants. The targeted sequencing sampled a wider range of pig diversity that whole-genome sequencing and, therefore, the rare variant calls could also represent genuine rare variants. However, with the depth of sequencing and the absence of stop-gain and frameshift indel variants in the whole-genome sequencing data, we are confident that there are no natural stop-gain variants and likely no natural frameshift indel variants in *CD163* in the evaluated pigs.

## Conclusions

We performed a deep survey of sequence variation in the *CD163* gene in domestic pigs. We found no potential knockout variants. *CD163* was moderately intolerant to variation, and showed evidence of positive selection in the pig lineage, but no evidence of selective sweeps during breeding.
